# Age-Related Reliability of B-Mode Analysis for Tailored Exosuit Assistance

**DOI:** 10.3390/s23031670

**Published:** 2023-02-03

**Authors:** Letizia Gionfrida, Richard W. Nuckols, Conor J. Walsh, Robert D. Howe

**Affiliations:** 1 Harvard John A. Paulson School of Engineering and Applied Sciences, Harvard University, Science and Engineering Complex, 150 Western Ave, Boston, MA 02134, USA; 2 Department of Systems Design Engineering, University of Waterloo, University Ave W, Waterloo, ON N2L 3G1, Canada

**Keywords:** wearable device, exoskeleton, muscle dynamics, b-mode ultrasound, aging, neural networks, fascicle length, muscle architecture

## Abstract

In the field of wearable robotics, assistance needs to be individualized for the user to maximize benefit. Information from muscle fascicles automatically recorded from brightness mode (B-mode) ultrasound has been used to design assistance profiles that are proportional to the estimated muscle force of young individuals. There is also a desire to develop similar strategies for older adults who may have age-altered physiology. This study introduces and validates a ResNet + 2x-LSTM model for extracting fascicle lengths in young and older adults. The labeling was generated in a semimanual manner for young (40,696 frames) and older adults (34,262 frames) depicting B-mode imaging of the medial gastrocnemius. First, the model was trained on young and tested on both young (R^2^ = 0.85, RMSE = 2.36 ± 1.51 mm, MAPE = 3.6%, aaDF = 0.48 ± 1.1 mm) and older adults (R^2^ = 0.53, RMSE = 4.7 ± 2.51 mm, MAPE = 5.19%, aaDF = 1.9 ± 1.39 mm). Then, the performances were trained across all ages (R^2^ = 0.79, RMSE = 3.95 ± 2.51 mm, MAPE = 4.5%, aaDF = 0.67 ± 1.8 mm). Although age-related muscle loss affects the error of the tracking methodology compared to the young population, the absolute percentage error for individual fascicles leads to a small variation of 3–5%, suggesting that the error may be acceptable in the generation of assistive force profiles.

## 1. Introduction

Wearable robotic devices have demonstrated the potential for assisting and augmenting human locomotion in a variety of domains, particularly clinical rehabilitation [1]. The effectiveness of exoskeleton systems relies on their capacity to address the heterogeneous needs of different users, while seamlessly adapting to diverse activities in real-world applications [2]. Parameters such as individuals’ age, muscle strength, and underlying musculoskeletal conditions must all play a role in how wearable assistive strategies are designed, implemented, and deployed [3]. To address these needs and fully harness the potential of assistive technologies in aging, the requirement for adaptation to diverse populations must be addressed.

Reduced mobility [4], diminished physical function and independence [5], and an increased risk of falls [6] in older adults are consequences of functional changes related to the strength of the lower extremity muscles [7]. Studies have shown that the major risk factor for falls in older adults is related to lower limb muscular weakness [8], due to muscle composition changes as people age [9]. In particular, changes in muscle quality due to aging are linked to variations in muscular architecture, such as fascicle length [10]. As a result, exoskeleton device design should carefully consider the age-related loss in muscle quality causing a lower capacity to generate muscle force [11] when designing human-in-the-loop assistive strategies [12].

Studies have shown that embracing assistive devices in the real world relies on customizing the assistance to the individual physiological responses [2,13,14]. One way to measure the individual physiological response during exoskeleton-assisted walking is through detecting muscle force generation directly measured using brightness-mode (B-mode) ultrasound to detect the generated force of a muscle and customize the assistive device for the individual [12,15]. B-mode imaging has increasingly become the gold-standard measurement to noninvasively quantify in vivo muscle dynamics, differentiating muscle architecture regions [16] and estimating muscle force production [17]. In particular, our previous investigation demonstrated that it is possible to extract muscle-based assistance (MBA) profiles from offline analysis of B-mode ultrasound images to estimate and design closed-loop assistance profiles to be proportional to the estimated muscle force [13]. While the adoption of this imaging modality has proven successful for younger adults [13,18,19], the practicability of adopting this tool in the older population, where muscles are smaller and have less well-defined fascicles remains to be determined.

A few investigations have validated the use of B-mode imaging as a valuable tool to extract muscle architecture in older adults [11,20], emphasizing the drawback of the time-consuming process of manual identification of fascicles. B-mode measurements of the muscle architecture have been traditionally quantified by manually finding the fascicle between the inner and outer aponeurosis [21,22]. However, manual identification of individual fascicles on multiple image frames can be extremely time-consuming [23,24,25].

Automatic imaging modalities have emerged, initially focusing on offline fascicle identification based on pixel intensity to assess the fascicle’s length and orientation [26,27], [28], but such techniques can be affected by image quality [29]. Semiautomated algorithms, such as detecting optical flow, present errors in the presence of large movements [30] and can increasingly drift over time [25,31], which produces unreliable tracking [32]. More recently, machine leaning-based ultrasound imaging techniques have emerged [16,33,34]. However, a preliminary attempt to determine muscle architecture from B-mode using a more generalizable convolutional neural network (CNN) ignored the temporal B-mode states and was not real-time [16]. This suggests that given the sequential nature of the continuous muscle recording present in ultrasound imaging, a deep learning model that can process sequential data would be preferred. Moreover, these automated approaches have not been validated in older populations, and the practicability of adopting an automated approach in older populations remains to be determined. Age-related changes in muscle quality may vary the utility of these automated approaches and the validity of these models is unknown, particularly when exoskeleton-assisted interventions are based on physiological parameters extracted with B-mode.

**Figure 1:**
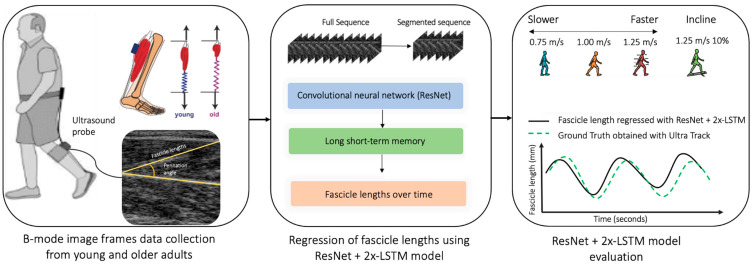
The proposed study where an ultrasound probe is placed over the medial gastrocnemius muscles, the proposed ResNet + 2x-LSTM architecture extracts the fascicles, and then the performances of the model are evaluated across different walking speeds of 0.75, 1.25, and 1.5 m/s and tasks (level ground and incline).

The purpose of this study is to introduce and evaluate an automated approach for extracting fascicle lengths in young and older adults (Figure 1). The key contributions are:

(a) A ResNet model [35] coupled with long short-term memory (LSTM) units trained and tested to reliably regress fascicle lengths from continuous ultrasound video recordings of young and older adults;
(b) A methodology able to process videos of arbitrary length, containing different populations (young and older adults), walking tasks (incline and decline), and walking velocities;
(c) A fast architecture that lays the foundations for a real-time model capable of executing tasks reliably in real-world scenarios for an individualized profile in human-in-the-loop assistive interventions.

The key novelty of the presented approach includes the validation of a real-time convolutional LSTM that outperforms previous investigations in terms of accuracy in both young and older adults.

To deliver these contributions, we proceed as follows. In Section 2, the experimental setup and the pre- and postprocessing of the implemented architectures are explained. Section 3 discusses the results when estimating fascicle length and pennation angles in young and older adults with the proposed methodology. The paper concludes with a discussion and conclusion, in Section 4 and Section 5, of how these results can be applied to individualize exoskeleton assistance to deliver tailored interventions to older adults.

## 2. Materials and Methods

In this section, B-mode ultrasound image recordings of the muscle fascicles, collected from young and older adults, were first segmented and labeled and then used to train two neural networks to identify muscle fascicle lengths. The labeling approach involved the estimation of fascicle lengths using the affine optical flow algorithm of UltraTrack [25] on smaller subsegments of the entire B-mode recordings. One single fascicle length (label) assigned to a given B-mode ultrasound image was used to train a neural network and enable fascicle length prediction from B-mode ultrasound images. We adopted a supervised learning strategy for the proposed neural network, which involved giving the neural networks labels. The inputs to the models were the image-label pairings obtained following a semiautomated labeling approach to identify fascicle lengths. Then, one model was trained only on data from young adults and tested on young and older adults, while the other was trained on both populations’ data and validated to detect muscle fascicles in young and older adults.

### 2.1. Participants

A total of nine young participants [n= 9, 3 females and 6 males; age = 29.1 ± 4.04 years (mean ± SD)] and four unimpaired older adults [n = 4, 1 female and 3 males; age = 75.6 ± 6 years (mean ± SD)] participated in the study.

All participants reported no previous history of physical or neurological impairment or disabilities in walking. Inclusion criteria were no severe cognitive deficits [Mini-Mental State Examination score > 23] [36], one or no falls in the previous months, no major surgery, no chronic pain or undergoing physical therapy, and no difficulties in daily activities or walking. The study was conducted according to the guidelines of the Declaration of Helsinki and approved by the Harvard Longwood Campus Institutional Review Board, protocol number IRB14-3608s. All methods were carried out in accordance with the approved study protocol and written informed consent prior to the start of the study was obtained for all participants.

### 2.2. Experimental Procedure and Study Design

Each participant walked on a treadmill for four walking tasks, comprising level ground walking at speeds of 0.75, 1.25, and 1.5 m/s and on a 10% incline at 1.25 m/s. The heel-strike timestamps were detected using an instrumented treadmill (Bertec, Columbus, OH, USA; 1200 Hz) to identify ground reaction forces (GRF). Participants walked while a low-profile ultrasound transducer (MicrUs, Telemed, Vilnius, Lithuania) was attached to the left leg. The ultrasound scan captured B-mode ultrasound images at a 113 Hz frame rate at 512 × 512 resolution (width and height) of the medial gastrocnemius muscle and soleus muscle. The MicrUs Telemed software allowed us to adjust the ultrasound image brightness and to normalize across participants during data acquisition. Subjects were instrumented with a 75 mm wide ultrasound probe with a 6 MHz center frequency. For the ultrasound probe, we picked a location over the medial gastrocnemius proximal to the gastrocnemius muscle–tendon junction such that aponeuroses of the gastrocnemius and soleus were approximately straight lines. As much as possible, the probe was adjusted to ensure that the fascicles were parallel straight lines and could be observed from one aponeurosis to the other. The probe was attached using self-adhesive athletic tape (Coban) to guarantee that it was almost the same for different subjects.

**Figure 2:**
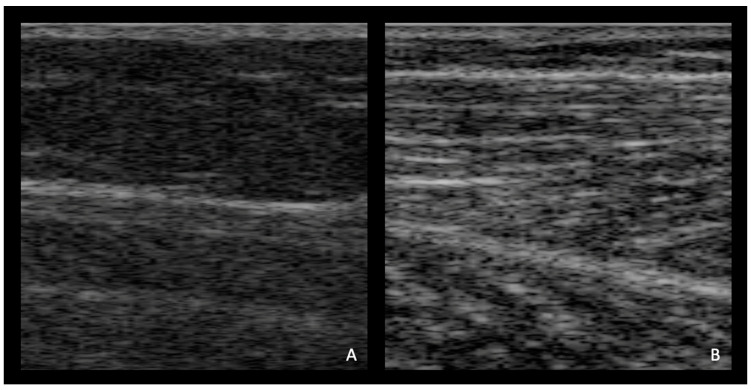
Randomly selected representative ultrasound images for (**A**) older adults and (**B**) young adults at 0.75 m/s 0.0° level ground.

Image frames included a representation on a sagittal plane of the upper and lower aponeurosis, the soleus muscle, and the medial gastrocnemius muscle (Figure 2). The dataset consisted of a total of 74,958 image frames: 40,696 collected from young participants and 34,262 collected from unimpaired older adults. We extracted one single fascicle length for each image frame.

### 2.3. Labeling

**Figure 3:**
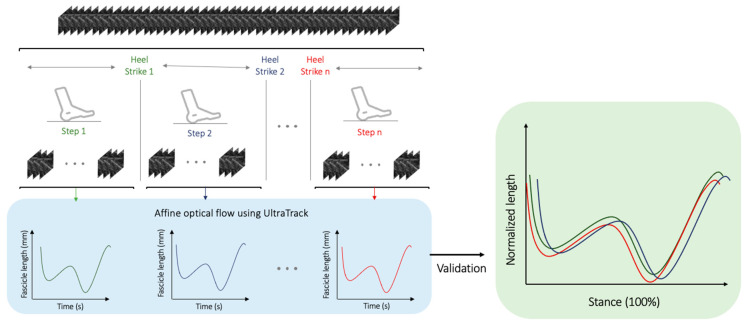
Illustration of high-level pipeline showing labeling used in this study. The continuous B-mode ultrasound recordings are segmented based on prerecorded heel strikes. The individual chucks are then processed using the affine optical flow algorithm of UltraTrack [25] to extract the fascicle lengths in millimeters (mm) across time in seconds (s) (in the lower left box). Each segmented sequence has many fascicle lengths (on average 130 frames in each segmented sequence), which form the fascicle lengths over time waveforms (left blue box). Finally, to validate the accuracy of the labeling methodology, the extracted individual segments of fascicles are plotted on top of each other over the stance phase (in the right box) following the approach proposed by Lai et al. [37].

Manual labeling (e.g., manual fascicle length identification for a single B-mode image) was combined with semiautomated fascicle length identification using UltraTrack [25] (Figure 3). Each sequence of a participant walking was segmented based on prerecorded heel strikes for a total of nine sequences on average for young and fourteen sequences on average for older adults across all walking tasks.

The segmentation into the smaller session was performed to minimize the spatial drift brought on by the affine optical and to mimic the gold-standard manual processing techniques in a sustainable manner. Each sequence of participant walking, containing on average 1441 image frames, was segmented into 130 image frames on average (chucks). The extracted chunks, segmented based on GRF heel-strike timestamps, were plotted using the methodology suggested by Lai et al. [37] to ensure correct labeling (Figure 3).

### 2.4. Model Design

Considering the nature of the image sequences as visual time series, we used a long-term recurrent convolutional network (also known as convolution LSTM or CNN + LSTM) [38,39]. As input, the model took in the 512 × 512 grayscale image frames and the time series labeled fascicle lengths to perform fascicle length detection. The architecture was composed of convolutional layers to perform local feature extraction and regression tasks. As CNNs have no memory of the input, the added LSTM components enabled both spatial and temporal inference, specifically designed for sequence prediction (e.g., order of image frames) with spatial inputs (e.g., 2D structure or pixels in an image).

**Figure 4:**
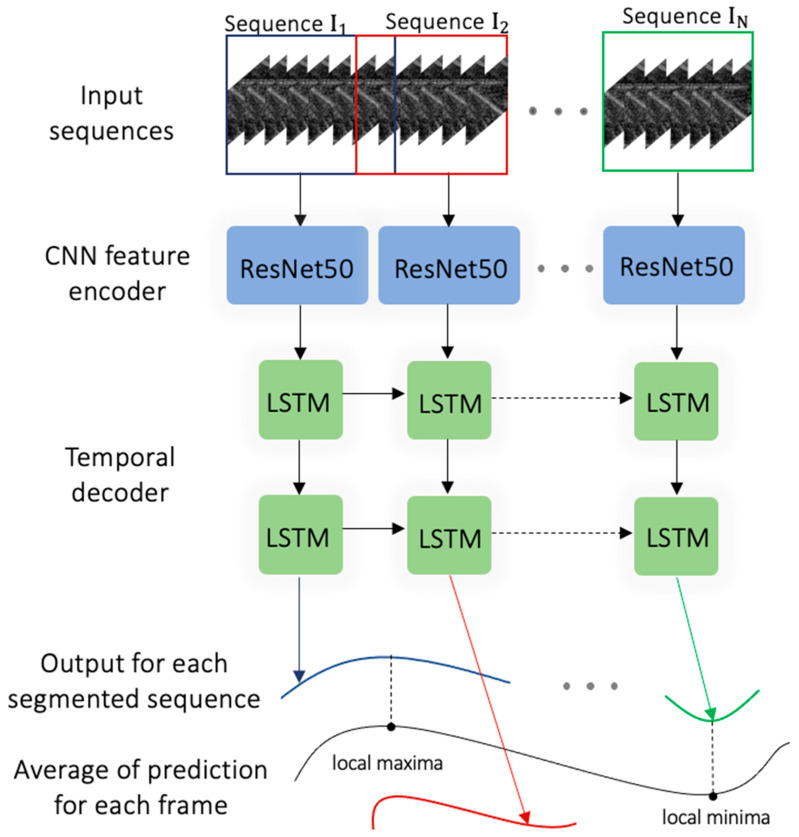
Schematic of the proposed convolutional neural network with two long short-term memory (LSTM) units. The individual segmented sequences that are input to the ResNet model [35] are then processed by two layers of LSTM units. Finally, the sliding window method processed fixed, overlapped, chunked sequences, generating multiple predictions for each frame. Once the local minima and the local maxima are identified for overlapping sequences, the average prediction fascicle length is then reconstructed from each subsegment.

The network architecture comprises (i) a convolutional unit for encoding the spatial information for each image frame of the ultrasonographic videos in input, (ii) LSTM units to decode the temporal information, and (iii) a regression unit for the prediction of the fascicle lengths of interest (Figure 4). The convolutional unit was used to extract a spatial feature vector from every image frame of the gastrocnemius in the image sequence. A state-of-the-art architecture was employed for the CNN unit, known as ResNet50 [35]). As ResNet50 was only capable of handling single images, transforming input pixels into a vector representation, two LSTM units were used to process the image features. The output of each LSTM unit was treated as input to the next unit. Finally, the output of the LSTM unit was regressed to predict the location of fascicles in individual image frames and their lengths. The model returned a time series prediction for each recording.

A sliding window method was used to allow the model to interpret video inputs of any duration. The adopted sliding window, as illustrated in the picture sequence, had overlapping chunks of a predetermined duration based on heel strikes. The neural network model was then fed to each segment, and a prediction vector $p_{k}$ was returned. The ultimate target output is determined as

$${\hat{y}}_{t}=\frac{1}{K}\overset{K}{\underset{k-1}{\sum }}p_{k,t}$$

where $p_{k,t}$ is the prediction for frame t in the kth segment, and K is the total number of predictions available for each frame, obtained from overlapping segments. A peak detection algorithm then searched for the local maxima and minima, representing the fascicle lengths during the gait cycle.

### 2.5. Model Evaluation

The model was implemented using the TensorFlow 2.0 deep learning framework [40] and trained on a local GPU. The loss function was the mean squared error (MSE) with the Adam optimizer initialized with a learning rate of 10^−5^. The dataset was divided into a 70/30 ratio for training and testing, respectively. The training was executed on 50 epochs, but early stopping was employed when needed to avoid overfitting, so training continued until the validation loss plateaued.

The ResNet + 2x-LSTM model was initially trained and tested on young participants (young-age-trained model), splitting the dataset for each participant at each walking task in a manner that both the training and testing were applied to the nine participants, but on different walking tasks, with 28,488 image frames used for training and 12,208 image frames used for testing. This model, pretrained on young adults (young-age-trained model), was also tested on the older adult population to evaluate the generalizability of a generic architecture trained on young adults and its effectiveness on older adults. The second test involved the ResNet + 2x-LSTM model training on the entire dataset containing both young and older adults (all-age-trained model), with 52,471 image frames used for training and 22,487 image frames used for validation. In both instances, data augmentation to prevent overfitting was used. In particular, spatial cropping between 0 and 10 pixels along each axis and rotation between −10° and 10° was adopted.

The inference metrics were the coefficient of determination (*R*^2^), root-mean-square errors (RMSEs) in mm of the fascicle length across participants for the two age groups, and the mean absolute percentage error (MAPE) with respect to the total fascicle length. As an additional endpoint, we measured the difference between each labeled target $y_{t}$ and the timestep prediction ${\hat{y}}_{t}$ as the average absolute frame difference (aaFD) for each segmented sequence, measured as

$$aaFD=\frac{1}{N}\overset{N}{\underset{t=1}{\sum }}\left|y_{t}-{\hat{y}}_{t}\right|$$

where *N* was the number of events for frame t within the test dataset. Finally, we also recorded the inference time for the network to output individual segmented fascicle lengths.

## 3. Results

### 3.1. Young-Age-Trained Model

The ResNet + 2x-LSTM trained and tested on young participants showed a high level of correlation of R^2^= 0.85. The RMSE across tasks for the two age groups was 2.36 ± 1.51 mm (mean ± SD). To keep a sense of proportion of the actual error with the respect to the total fascicle length, the MAPE reported how much these errors meant with respect to the total length of the fascicles, presenting a value of 3.69% on average with respect to the total fascicle lengths. The average error reported for each subsegment inferred (aaFD) was 0.48 ± 1.10 mm (mean ± SD). The same architecture was also tested on older adults to evaluate the model’s effectiveness to extract fascicle lengths from a population not seen in testing. The coefficient of determination was moderate (R2= 0.53), with an increased RMSE of 4.7 ± 4.66 mm (mean ± SD), MAPE = 5.19%, and aaFD = 1.9 ± 1.39 mm. The network average detection time was 0.59 ± 1.53 s (s) for detecting each segmented event. A summary of the R^2^, RMSE, MAPE, aaFD, and average detection time for this first young-age-trained model is in Table 1. To improve the performance of the model, the architecture was retrained on the young and older adult image frames (combined).

### 3.2. All-Age-Trained Model

**Figure 5:**
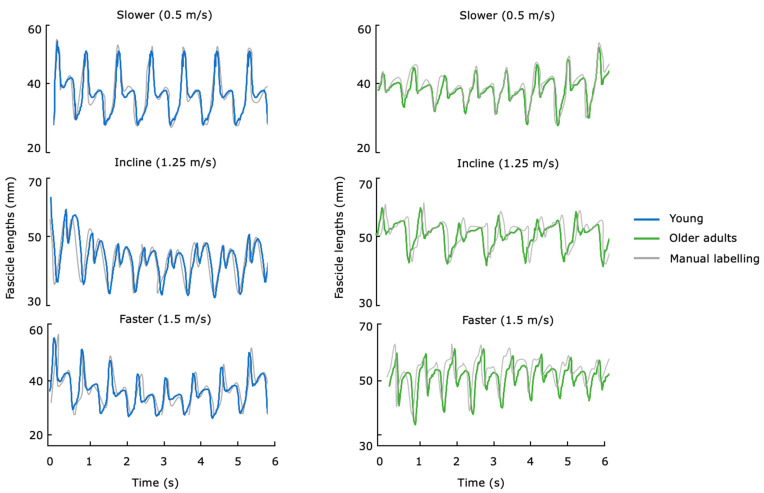
Fascicle lengths (in millimeters) versus time (in seconds) in output from the ResNet + 2x-LSTM algorithm for the all-age-trained model for both young and older adults compared against the (semiautomated) manual labeling. The time series data show the trends across three tasks, including level ground at walking speeds of 0.75 m/s (slower), 1.25 m/s at 10% incline, and 1.5 m/s (faster) of the muscle fascicles regressed from the B-mode ultrasound images.

**Figure 6:**
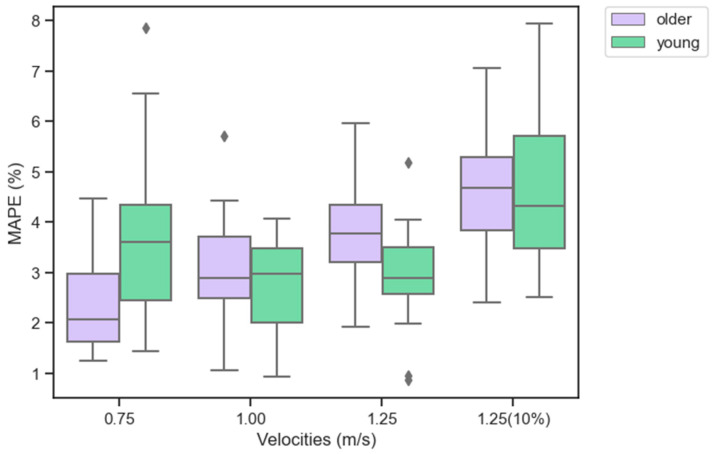
Illustration of the mean absolute percentage error (MAPE) in percentage (%) for the fascicle lengths across participants (young in light green and older in light purple) across the four task velocities (level ground at walking speeds of 0.75, 1.25, and 1.5 m/s and 10% incline at 1.25 m/s). The results represent the training performed across participant ages using the ResNet + 2x-LSTM introduced in the study. The box plots extend from the lower (25th) to upper (75th) quartile values of the data, with a line at the median value. The whiskers (vertical lines) extend from the box to show the range of the data, from the minimum to the maximum value. Outliers (data points located outside the whiskers of the box plot and numerically distant from the rest of the data) are represented as black dots.

The ResNet + 2x-LSTM was then retrained and validated with young and older adult image frames combined. The model presented an increased coefficient of determination value of 0.79 and an error of 3.95 ± 2.51 mm. The MAPE for older adults was 4.5% and for the subsegment was aaFD = 0.67± 1.8 mm. The proposed retrained ResNet + 2x-LSTM presented an average inference time of 0.62 ± 0.32s for detecting each segmented event. A summary of the R^2^, RMSE, MAPE, aaFD, and average detection time for this second training is also illustrated in Table 1 (as the results of the young adults did not improve with further training, only the results of the older adults are reported for the all-age-trained model). Figure 5 shows fascicle lengths in output for young and older adults across tasks. To further investigate the model across velocities and tasks, the MAPE for the two age groups at different walking tasks is illustrated in Figure 6.

The average time to manually annotate fascicle lengths to generate individual B-mode ultrasound recordings was 56 ± 13 s (mean ± SD) per segmented fascicle length. The equivalent time performed by the ResNet + 2x-LST demonstrated an advantage, having an inference time of 0.62 s for detecting each event.

## 4. Discussion

This study sought to investigate the feasibility of a neural network to automatically extract fascicle lengths in young and older adults. Fascicle length estimates can be used to generate profiles tailored to the person in human-in-the-loop assistive strategies. These strategies have been used on young adults and have the potential to be useful for older adults who have declines in muscle mass, particularly in the lower extremities [11,20,41]. In automated methods to extract muscle fascicles from B-mode, the literature has mainly focused on young adults, and to the best of our knowledge, no investigation has specifically addressed the error of automated tracking methods for individualized assistance in older adults. We demonstrated (1) the error of an architecture made of a ResNet model combined with LSTMs for both young and older adults; (2) the generalizability of the all-age-trained model in terms of tasks, population, and arbitrary video length; (3) the processing speed that supports physiology-based assistance.

The error of the ResNet + 2x-LSTM algorithm decreased in the all-age-trained model for older adults but stayed the same in the two tests for young adults. This was likely due to the reduced fascicle contrast in older adults, which could result in a more challenging detection task for the model. Although the error of the tracking for older adults increased compared to the young population, the average fascicle length across participants and tasks was found to be 54.5 mm; therefore, the variation of 2 mm is small, and may lead to errors of 3 to 5% in the generated force profiles [13].

This study also addressed the shortage of ultrasonographic training data by developing a sliding window approach for the ResNet + 2x-LSTM algorithm. This approach enabled the inference of smaller subsegments of fascicles of arbitrary lengths. Although the sliding window is a very popular technique for training processes on CNN [42,43], the novelty of this research is the combination of the approach to multiple LSTM units for fascicle length extraction. While the proposed model was trained on fixed-length sequences, no a priori information was provided in input to the model on the length of the inputs, suggesting that the model would successfully deal with varying-length inputs and maintain good performances across participants and tasks. An interesting future direction would be a segmentation model to automatically obtain the optimal window size for different ultrasound imaging recordings in terms of recognition, speed, and accuracy.

The time for our automated model, executed on the GPU, was significantly faster than the human-led process based on the affine optical flow to manually annotate fascicle lengths. A useful direction for the development of an image analysis interface would involve a web-based, interactive, real-time platform, which could significantly speed the annotation process.

This study comes with some limitations. First, we could not analyze our data based on gender because of the small number of subjects in each class. Second, we acknowledge the class unbalances related to the number of subjects present in each cluster. However, the number of frames was almost the same due to longer recordings in the older adult population. Third, partial manual fascicle detection was used to train the model using chunks processed via Ultra Track; intrinsic errors due to affine optical flow could have caused discrepancies in the model. However, given the amount of training data, the performance of the proposed ResNet + 2x-LSTM algorithm is well positioned to further evaluate it against other gold-standard tracking methods (manually) on young and older adults.

In the future, it would be interesting to evaluate the performances of the proposed model for real-time exoskeleton assistance. Muscle-based assistance may be able to enhance assistance methodologies and closed-loop controllers to allow real-time dynamic control for real-world tasks across different populations. Exoskeleton device design should carefully consider the age-related loss in muscle quality causing a lower capacity to generate muscle force [11,44,45]. Studies have shown that the major risk factors for falls in older adults are related to lower limb muscular weakness [8], due to muscle composition changes as people age [9]. In particular, changes in muscle quality due to aging are linked to variations in muscular architecture such as fascicle length [10]. This has particular relevance for the design of exoskeleton devices that should consider the age-related loss in muscle quality, suggesting that the factor introduced by age differences in muscle architecture can be considered acceptable when designing human-in-the-loop assistive strategies using in vivo muscle dynamics. This represents a great potential advantage of the imaging modality, and the proposed methodology, for real-time assistance. The approach presented here may be relevant for the design of strategies for fall prevention in older adults.

## 5. Conclusions

This paper introduces a method for autonomous fascicle length estimation from B-mode ultrasound in young and older adults. The experimental results illustrate that the proposed technique can effectively estimate the desired parameters for the two populations. This research can facilitate the adoption of lower-extremity assistive exoskeleton devices, addressing the need for assistance profiles tailored to the participant’s needs, specifically adjusted to the older adult populations. This validation for lower-extremity exoskeletons may have the potential to improve the physiology-driven applications and support real-world adoptions for adults with limited mobility during walking.

## Figures and Tables

**Table 1:** Comparison of the performances of the two proposed ResNet + 2x-LSTM algorithms for young-age-trained and all-age-trained models. The table indicates the value of the coefficient of determination (*R*^2^), the root-mean-square error (RMSE) in millimeters (mm), the mean absolute percentage error with respect to the total length of the fascicles (in %), the average absolute frame difference (*aaFD*) of the individual subsegments in mm, and the average detection time (mean in seconds ± SD).

Performance Metrics	Young-Age-Trained		All-Age-Trained
	Young	Older	Older
R^2^	0.85	0.53	0.79
RMSE (in mm)	2.36 ±1.51	4.7 ± 4.66	3.95 ± 2.51
MAPE (%)	3.69	5.19	4.5
aaFD (in mm)	0.48± 1.10	1.9 ± 1.39	0.67± 1.8
Average detection time (s)	0.59 ± 1.53		0.62 ± 0.32

## Data Availability

Not applicable.
